# Design and directed evolution of genetically encoded cGMP sensors

**DOI:** 10.1186/2050-6511-16-S1-A48

**Published:** 2015-09-02

**Authors:** Arne Fabritius, Oliver Griesbeck

**Affiliations:** 1 Max Planck Institute of Neurobiology, Martinsried, Bayern, Germany

## 

Imaging cGMP transients in in vivo models is very challenging and therefore requires probes that are highly optimized for this task. Several key factors are to be considered while developing such a biosensor: affinity and dynamic range – to cover physiologically relevant concentration ranges, sensitivity – to facilitate meaningful imaging of small variations in concentration, and overall brightness and stability – to increase signal/noise ratio and provide comparable results through a variety of compartments and tissues.

We seek to generate a robust genetically encoded FRET-sensor for cGMP imaging. Our approach adapts protocols that our lab developed and has already successfully applied in the development of Ca^2+^ biosensors [1,2]. This method combines an initial rational design approach with several iterations of directed evolution. Rational design of these probes supplies prototypes, which are turned into partially randomized libraries. These libraries are subsequently screened in *E.coli* by means of a semiautomatic high throughput process. Promising candidates are biochemically characterized in vitro and later on tested in cell culture.

Initial results already show substantial improvements over existing cGMP biosensors and indicate, that the success in the field of Ca^2+^ imaging can be repeated for cGMP.
